# Involving elderly research participants in the co-design of a future multi-generational cohort study

**DOI:** 10.1186/s40900-021-00271-4

**Published:** 2021-05-03

**Authors:** Jack S. Nunn, Merrin Sulovski, Jane Tiller, Bruce Holloway, Darshini Ayton, Paul Lacaze

**Affiliations:** 1 School of Psychology and Public Health, La Trobe University, Melbourne, Victoria Australia; 2 Director of Science for All (Education Charity registered in Australia), Melbourne, Australia; 3 Department of Epidemiology and Preventive Medicine, School of Public Health and Preventive Medicine, Monash University, Melbourne, Australia

**Keywords:** Co-design, Multi-generational, Elderly, Participatory, Standardised reporting, Dementia, STARDIT, Case study, Genomics

## Abstract

**Background:**

It has been proposed that the existing ASPirin in Reducing Events in the Elderly Extension observational cohort study (ASPREE-XT) would provide a platform for a future multigenerational research study (MGRS). An advert was sent to 14,268 participants (aged 74 years and older, from Australia, and located in both metropolitan and rural locations) to invite them to share views and preferences about being involved in the co-design of a future MGRS, as their preferences were not known. The objective of this article is to report as a case study the process of involving study participants and how this impacted the co-design of a proposed multi-generational research study, using a novel standardised reporting tool.

**Methods:**

We used participatory action research to involve elderly research participants in the co-design of a proposed multi-generational cohort study between 2017 and 2019 using newsletters, telephone interviews and an in-person workshop. We used the novel ‘Standardised Data on Initiatives Alpha Version 0.1’ (STARDIT 0.1) to plan and report how participant involvement activities positively impacted the study design.

**Results:**

Fifty-nine ASPREE-XT participants were interviewed by telephone and 18 participants attended a face-to-face event. Involving participants positively impacted the proposed study design by improving the research objectives, developing protocols, influencing funding decisions and improving ethics applications. Learning points included the importance of maintaining the ideals of ASPREE-XT (respect, quality and transparency); research participants’ preference for the option of receiving results (including genetic results); participants’ need for involvement in decisions about recruitment, data access, governance and other ethical issues; and the preference for different communication methods, including both face-to-face and online methods. Data from the process indicated it was highly valued by all stakeholders, including research participants, study staff and lead investigators. Involvement of participants was described by a lead study investigator of ASPREE-XT as “enormously helpful”.

**Conclusions:**

This case study demonstrates that including participants in the design of a research study positively impacted the study design, participants and researchers. Using a standardised reporting tool to describe the methods and impacts provides a way for learning from this case study to inform future research studies planning to involve people.

**Supplementary Information:**

The online version contains supplementary material available at 10.1186/s40900-021-00271-4.

## Plain English summary

Research which follows a population of people over time is a valuable way to learn about what contributes to both wellness and disease. Some studies of this kind look at multiple generations and such studies have had significant positive impacts on public health. However, such studies are challenging to establish and expensive to maintain.

It has been proposed that the existing ASPirin in Reducing Events in the Elderly Extension study (ASPREE-XT) would be a good basis for a future multigenerational research study (MGRS). There is evidence that involving potential participants in co-designing research can improve the quality of the study design, recruitment and acceptability of the research. A team of people including a current ASPREE-XT participant were involved in planning how potential participants would be involved in the co-design process. An advert was sent to 14,268 participants of the ASPREE-XT observational cohort study to invite them to be involved in the process of planning a future multi-generational research study.

Fifty-nine ASPREE-XT participants were interviewed by telephone and 18 attended a face-to-face workshop event. We used a newly developed standardised format to plan and report how participant involvement activities positively impacted the study design (Standardised Data on Initiatives - Alpha Version 0.1).

Involving participants positively impacted the proposed study design by improving the research objectives, developing protocols, influencing funding decisions and improving ethics applications. This case study provides evidence that including participants in the design of a research study positively impacted the study design, participants and researchers.

## Introduction

The ASPirin in Reducing Events in the Elderly (ASPREE) trial (2010–2018), a randomised controlled trial for aspirin in healthy older people, recruited 19,114 participants from Australia and the USA [1–4]. The median age at recruitment was 74 years. The ASPREE Healthy Ageing Biobank is a sub-study which has collected biospecimens from over 15,000 ASPREE participants, alongside detailed medical records, lifestyle, cognitive function and physical testing data [5]. ASPREE researchers are conducting various types of genomics and biomarker research. ASPREE had a remarkably high retention rate, with only 1.2% of participants withdrawing from the study, with 90% still alive and attending annual visits after an average of 4.5 years follow-up [6, 7].

The ASPREE-XT (eXTension) is a follow up observational study that was established in 2018 to continue to collect data from ASPREE participants for another 5 years until 2024. ASPREE-XT participants are over the age of 74 (defined as elderly [8]) and Australian participants are located in both metropolitan and rural locations. Multiple stakeholders, including a participant advisor, proposed a new multi-generational research study (MGRS) which could recruit direct descendants of ASPREE-XT participants, as part of a longitudinal observational study alongside their relatives for two generations or more. MGRS are challenging to establish and expensive to maintain, yet their value to medical and epidemiological research is significant. ASPREE-XT has been proposed as a platform for a multi-generational study. Recruiting ASPREE-XT participants and their descendants to a MGRS would provide a large starting population by international standards (> 19,114).

Previous multi-generational, longitudinal cohort studies have had significant positive impacts on public health. Examples include the Framingham Heart Study [9], Lothian Birth Cohort [10] and Dubbo Osteoporosis Epidemiology Study [11], all of which had substantially lower starting populations than ASPREE-XT (19,114) [5]. ASPREE-XT provides a rare opportunity for such a study in Australia, already combining high-quality medical record data with genomic data on a large number of elderly Australians [5]. Additionally, the cohort is already well engaged, with all 59 surveyed participants supportive of a proposed MGRS.

ASPREE-XT’s unique focus on healthy ageing (with the starting cohort having no reported cognitive, cancer or cardio-vascular related health issues) adds substantial value to public health, medical, epidemiological and geriatric research. Previously collected genomic (including epigenetic) data, combined with detailed medical data and ongoing cognitive assessment, allow that a MGRS with the ASPREE-XT cohort would be of considerable value to science for decades to come, for example in dementia research [12]. The proposed study would examine health outcomes in a large, well-monitored cohort; and provide data to help inform our understanding about the genetic and environmental determinants of health and disease, across multiple generations. However, this is only possible if people choose to participate.

Many clinical research studies are underpowered due to poor recruitment and retention [13]. Involving participants and the public in research design has been shown to improve the recruitment [14, 15], quality and relevance of research [16, 17]. The concept of involvement is research being done ‘with’ people rather than ‘on’ them [18]. Involving the public, patients, research participants and other stakeholders in actively contributing to the research process can lead to a range of positive impacts and outcomes [19]. These impacts can include improving the recruitment [14], quality and relevance of research [16, 17].

In human genomics research, the need to involve the public and other stakeholders is a crucial aspect of responsible research practice [16, 20, 21]. The term ‘stakeholder’ here means anyone who has a ‘stake’ in the research, in particular those with important knowledge, expertise or views that should be taken into account [20, 22]. This includes ASPREE-XT participants, study staff and academic research investigators and the wider public. At the earliest stage of the research cycle (the conceptual stage), some current ASPREE-XT participants were invited to be involved in the co-design of a new MGRS.

The aim of this study was to report as a case study the processes of involving ASPREE-XT participants in the co-design of a proposed multi-generational research study, and how this impacted study design. The processes of involving people were guided by a participatory action research (PAR) paragidm [23] (p1). The research objectives were to involve potential participants in the co-design of a new MGRS; plan the process in a standardised way; identify themes and preferences; and evaluate then report the process using a standardised reporting tool [24]. In this article we aim to outline how people were involved in the co-design process in order to appraise the methodology. We hope the learning from article might inform future best-practice, and support those planning and evaluating involvement in research.

### Terminology

We have used consistent language in this paper to describe concepts such as ‘involvement’. To aid readers, Table 1 provides definitions of important terms used consistently throughout this paper.

**Table 1:** Definitions of terms

**Involvement** – The words **‘involvement’** or ‘being **involved’** describe the concept of people being ‘involved’ in research. This is when research is carried out ‘with’ people rather than ‘on’ them [18]. ‘Involvement’ can also be defined as when other people aside from the research team, such as the public, patients, research participants and other stakeholders, actively contribute to the research process [19]. It is the ‘active involvement’ in shaping and guiding research, rather than only providing data [25].	
**Participant** – a person who participated in the process of sharing views and perspectives about the proposed MGRS, including sharing views about preferences for any future involvement. The term ‘ASPREE-XT participants’ will be used when specifically referring to participants from the existing study.	
**Participant advisor** – before inviting people to become participants, it was necessary to involve a small number of participants to help advise and plan the process. These participants were chosen from an existing reference group of ASPREE-XT participants.	
**Stakeholder** – this term includes anyone who has a ‘stake’ in the research, in particular those who have important knowledge, views or perspectives that should be taken into account [20, 22]. In this paper it refers to participants, participant advisors and ASPREE-XT study team members (including researchers, ASPREE-XT participant assessors and lead investigators).	
**Study team** – this process was guided by the study team, who consisted of academic researchers, ASPREE-XT participant assessors, a participant advisor and a lead investigator.	
**The process** – this term will be used to describe the process of involving ASPREE-XT participants by inviting them to share views and perspectives about a potential future MGRS. This process includes the co-creation of this case study with participant advisors.	
**Participatory action research (PAR) -** this term describes a number of related approaches, including forms of action research which embrace a participatory philosophy and include ‘co-design’ and ‘co-production’ of research [23] (p1).	

## Materials and methods

### Study design

**Fig. 1:**
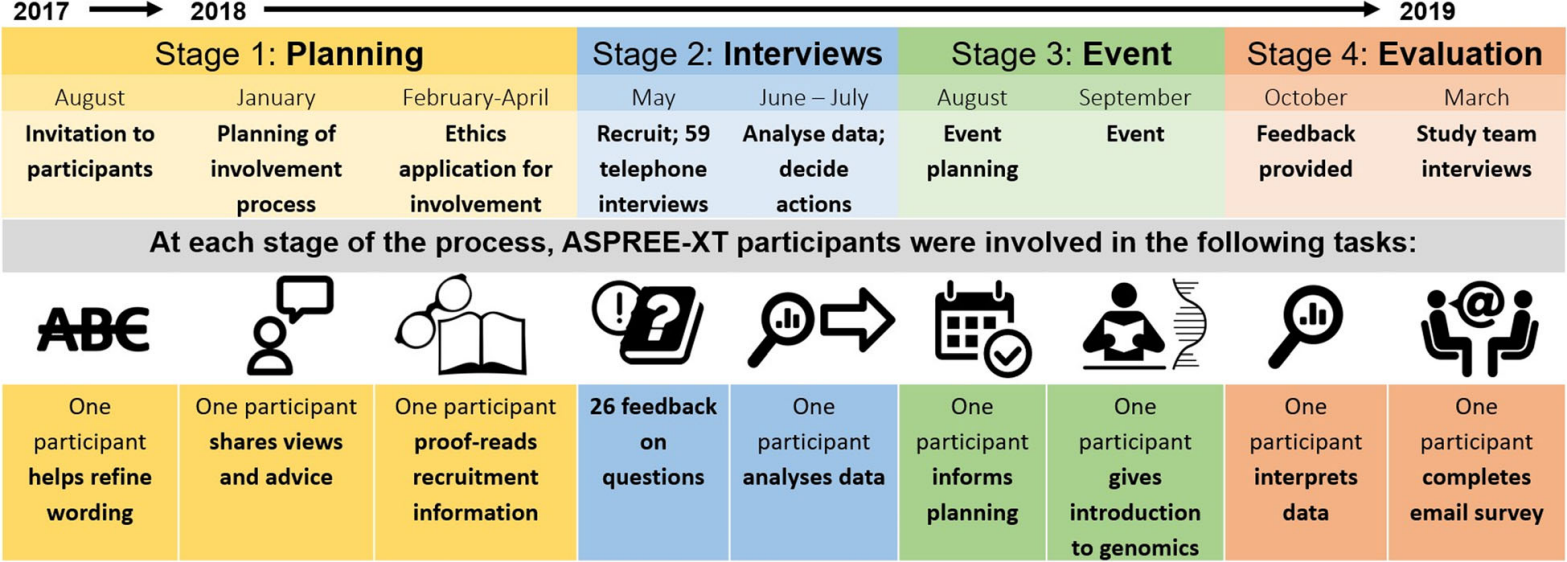
Process timeline of the participant involvement

A participatory action research (PAR) paradigm was chosen to guide the process with co-design and reporting guided by a number of frameworks [28–32]. PAR is an umbrella term which describes a number of related approaches, including forms of action research which embrace a participatory philosophy and include ‘co-design’ and ‘co-production’ of research [23] (p1). During the study design, we applied this co-design process where researchers and other relevant stakeholders (including research participants) “work together, sharing power and responsibility from the start to the end of the project” [33], including knowledge generation and translation [33, 34]. Figure 1 summarises the process we used (Fig. 1). The ASPREE participant advisor was an integral member of the study team, through each stage.

### Guiding paradigms and frameworks

The process was guided by a number of international PAR methodology frameworks [35–37], including the International Collaboration for Participatory Health Research [38]. The PAR method was also informed by an international review of involvement in genomic research carried out by some members of the study team [16]. Learning from this review informed the subsequent development and application of ‘Standardised Data on Initiatives - Alpha Version 0.1’ (STARDIT) [24]. STARDIT includes a tool to map people’s preferences for involvement in a standardised way, including mapping views on who should be involved and how. STARDIT was then used to guide co-design of the process, and to subsequently report how people were involved, using standardised data. The STARDIT framework was also used in parallel with a thematic analysis, which organised data into pre-defined ‘super-categories’ which allow consistent comparison with other data using this reporting framework (see Stage 4) [24].

We used a case study research methodology to record and describe the process of involving participants in the co-design of a proposed MGRS. Case study research methodology is a form of empirical inquiry. In this study it is presented as an instrumental case study, where the purpose is to understand the particular case and to provide data that could produce useful generalisations by using inferences from the data [39] (p109).

The selection of the population for this case study was informed by a number of factors which were appraised by the study team, including ethical, pragmatic and population considerations (see Stage 2) [40, 41]. One of the study team members (PL) is a lead investigator with the ASPREE-XT study, which was used as a starting point by the study team to explore the appropriateness of this case study.

### Data collection and reporting

The data collection and reporting was informed by a number of frameworks for reporting involvement in research, which included ‘Guidance for Reporting Involvement of Patients and Public’ (GRIPP2), the ‘Public Involvement Impact Assessment Framework Guidance’ (PiiAF), and the aforementioned STARDIT [20, 24, 28, 29, 37, 42–44]. Qualitative and quantitative data was collected from multiple sources, including meeting notes, audio recordings, documents, emails and surveys. Further information about how data sources were collected and analysed is in Table 2.

**Table 2:** Summary of data collection and analysis

Data source description	Analysis method
**Meetings** – including meeting notes, recordings and relevant documents	**Qualitative** (content analysis)
**Telephone interviews** – including recordings, notes made by interviewer, summary documents and related emails	**Qualitative** (thematic analysis, content analysis) **Quantitative** (number of responses)
**Event** – including audio and video recordings, interviews, written notes and feedback forms	**Qualitative** (content analysis) **Quantitative** (number of responses)
**Study team surveys** – responses to open ended questions by email	**Qualitative** (content analysis)
**Other data** – this included reflexive research diaries, relevant emails, financial and other relevant documents	**Qualitative** (thematic analysis) **Quantitative** (analysis of cost)

## Stages of research

### Stage 1: planning

The study team held four meetings to co-design the involvement activities. One participant advisor was involved in a number of tasks including reviewing and improving the written information, telephone interview questions, and the facilitation plan for the event. For example, the participant advisor recommended language was adapted from generic terms such as ‘using online tools’ to ‘using smartphones and computers’ in order to make sure it was comprehensible to the audience.

### Stage 2: recruitment and telephone interviews

The recruitment of participants was was opportunistic, and relied upon participants voluntarily responding to a brief invitation in a routine ASPREE study newsletter which is sent by mail to all ASPREE participants four times per year. Inclusion criteria for telephone interview participants was that they were ASPREE-XT participants. After discussion with ASPREE-XT staff and a participant advisor, ethical approval was granted to place an advert in a newsletter which was posted to the 14,268 ASPREE participants in June 2017. The process we used for recruiting and involving ASPREE-XT participants has been divided into four chronological stages, summarised above in Fig. 1 (Fig. 1).

Once people responded to the advert, the recruitment and consent process was completed and participants were interviewed by telephone (May 2018). A summary of the questions and responses from the telephone interviews is shown in Table 3. For a list of all questions asked, see Additional file 2. Participants were asked about their willingness to provide feedback throughout the study, and to be involved in study design, as well as preferences for modes of communication. The definition of involvement below used in the script was co-designed with participants for subsequent interviewees. Versions 1 through 1.3 were piloted with 15 participants, with this data included in the analysis (see Additional File 2).

**Table 3:** Summary of data from telephone interviews

Questions (closed)	Results	% (number)
1. Do you think participants like yourself should be involved in helping design research projects, or just researchers?	Just researchers	**39** (11/28)
	Participants involved	**46** (13/28)
	Unsure	**14** (4/28)
2. Would you be willing to provide feedback and share your views and perspectives by commenting throughout the research process?	Yes	**100** (32/32)
	No	**0** (0/32)
3. Would you like to be more involved in study design rather than just participating?	Yes	**65** (11/17)
	No	**12** (2/17)
	Unsure	**23** (4/17)
4. Would you be more or less likely to participate in research if participants were involved in design	More likely	**25** (2/8)
	Less likely	**0** (0/8)
	Unsure	**13** (1/8)
	Wouldn’t influence participation	**13** (1/8)
5. What is your preferred mode of communication for being involved	Face to face event	**25** (15/59)
	Post	**31** (18/59)
	Online discussion	**8** (5/59)
	Online questionnaire	**17** (10/59)
6. If genetic research results were made available, which types of genetic testing would you be interested in? ^a^	Medical	**97** (32/33)
	Ancestry	**97** (32/33)
	Drug response	**94** (31/33)
7. If only one option for genetic testing was available, which one would you prefer?	Medical	**65** (11/17)
	Ancestry	**12** (2/17)
	Drug response	**6** (1/17)
	No preference	**18** (3/17)

^a^Note that percentages indicate the percentage of participants who responded to that particular question with that response. On question six, participants could select multiple answers, so totals do not add up to 100%. The number in brackets represents (number of people with that response/ number of people asked that question)

Pre-question script: ‘Traditionally, research studies have been designed and conducted only by researchers, and people invited to participate. We are challenging this idea of researchers being the only experts.’

Question: Would you be willing to provide feedback and share your views and perspectives by commenting throughout the research process?’

Twenty relevant interviews were transcribed, coded and categorised (JN), with relevant interviews identified by two investigators independently (JN, PL) [45]. To reduce any unconscious selection bias, a sample of over 10% of the interviews (6/59) were selected at random and included in the analysis.

### Stage 3: event

Eighteen participants opted to attend a four-hour workshop event in central Melbourne, led by JN, who is an experienced facilitator. The event was co-designed by the study team, and was informed by interview data and international best-practices for involvement events [36, 46]. As well as offspring of participants, carers and loved-ones were invited to attend in order to offer practical and emotional support.

The event included an introduction to the proposed MGRS by the lead ASPREE-XT genomics researcher (PL); a plain-English introduction to genomics by the participant advisor, an expert in genomics (BH); a summary of the telephone interview results by the interviewer (MS); and an interactive session which included open questions about the types of information participants would like returned and recruitment of family members (JN).

The final session included a presentation and interactive discussion about involvement in research, led by the event facilitator (JN). This session explored preferences about how people would like to be involved, with open and closed questions. Questions included preferences about tasks and modes of communication.

Throughout the event, participants shared their views on a range of issues through interactive discussions, voting (by show of hands) and anonymous written feedback.

### Stage 4: evaluation and analysis

#### Evaluation and reflection

All members of the study team (except JN) were surveyed six months after the face-to-face event in order to integrate the valuable views and perspectives of those involved in co-designing and delivering the process. Design of surveys was informed by frameworks for planning and reporting public involvement [28, 29]. The study team were asked 11 questions in a survey (available in Additional File 1), and the data from the four surveys was coded and categorised (JN) using STARDIT [24].

### Data analysis

The stages of qualitative data analysis included data mapping and familiarisation; transcription; coding; searching for themes; reviewing themes with study team members; labelling and summarising themes; and reporting the findings [45]. In order to enhance validity of the analysis, two authors (JN, MS) independently analysed the data thematically, with the analysis then checked for validity (‘member checked’) by a third author (PL) [45, 47, 48]. Document and content analysis was also used, combining thematic analysis with quantitative analysis [44]. Standardised categories (STARDIT) [24] were used during content analysis of the data in order to facilitate comparison with other research projects [24, 44]. More information about the data sources is available in Additional file 1, along with a STARDIT report [49].

## Results

**Fig. 2:**
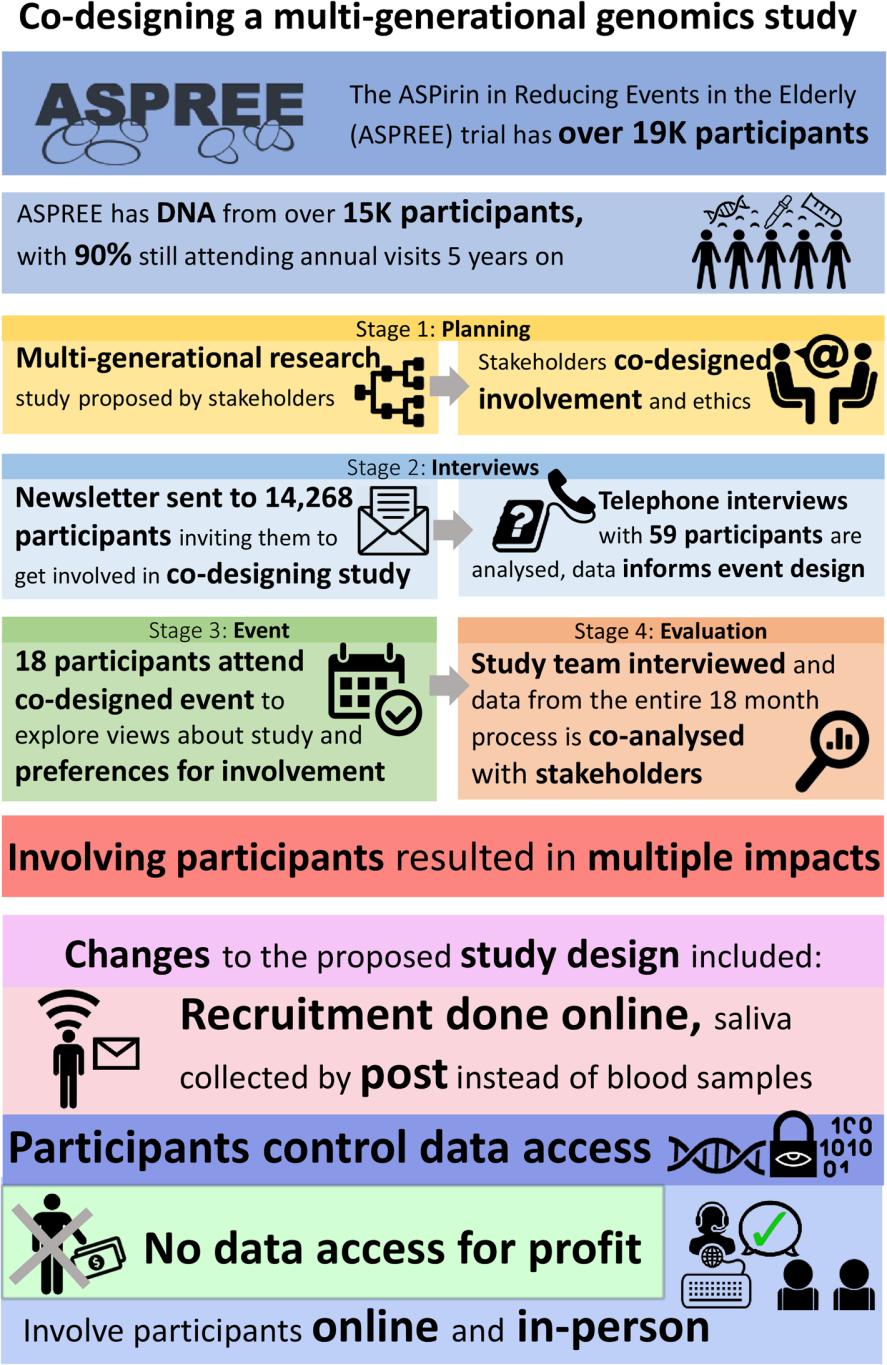
Co-designing multi-generational genomics research

The results are presented in three sections. Section one provides results from the stages outlined in Fig. 1 (Fig. 1), section two shares the perspectives of ASPREE-XT participants and other stakeholders, and in section three we describe how participant involvement impacted study design in seven ways. We quote participants directly, assigning each a unique number. Figure 2 summarises the entire process, results and impacts (Fig. 2). For quantitative data from the telephone interviews, see Additional File 3.

### Section 1: results from stages 1–4

#### Results from stage 1: planning

Data from the study team suggests that the input of the participant advisor during the early co-design stage had a number of benefits, in particular in identifying the best approaches for engaging the broader ASPREE population; improving the wording of participant information resources and improving question design for the interviews.

#### Results from stage 2: recruitment and telephone interviews

After reading the advert in the newsletter, 76 ASPREE-XT participants agreed to participate in the process. We interviewed 59 people by telephone, with the remaining 17 not responding to a number of follow-up calls. Participants were a mixture of people living in metropolitan and rural areas.

Interview participants expressed a strong interest in receiving results from genetic research. The strongest preference was for genetic results of medical significance (rather than ancestry or drug-response).

All telephone interview participants expressed willingness to provide feedback throughout the process of planning the proposed MGRS. While most participants understood the concept of involvement, 9 of the 20 analysed expressed self-doubt about how they could be involved. Of 20 analysed telephone interviews, five participants seemed unclear about what tasks they could be involved in, suggesting the concept of participant involvement was new to them. Six participants sought clarity about what was expected of them when being involved, or discussed the skills and knowledge required. Further, participants stated that the goals and processes of involvement needed to be clear in order to avoid “spending too much time” and were willing to be involved as long as the task had purpose and was not “just for the sake of chatting” (P1).

During the telephone interview stage, the interviewer initially “struggled to explain” the concept of involvement (as distinct from participation in an active study) (MS). Co-designing and making changes to the language used in the telephone interviews (version 1.5 onwards) appeared to improve participant understanding of the concept of involvement. Changes included providing a clearer definition and pre-question script and including actual examples of involvement (such as overseeing ethical decisions about data access), thereby avoiding jargonistic abstract concepts such as ‘involvement’.

#### Results from stage 3: event

Eighteen of the invited 76 participants attended the face-to-face event. Participants were a mixture of people living in metropolitan and rural areas, with some travelling for a number of hours in order to attend. When specific tasks were discussed at the event, 10 participants expressed interest in being involved in recruitment and communication tasks, 7 in data access decisions, and 11 in ethical decisions. When surveyed after the event, the study team reported that involving participants positively impacted the proposed study design by improving the research objectives, developing protocols, influencing funding decisions and improving ethics applications. Videos of the event discussions and interviews with participants will be shared in the public domain.

#### Results from stage 4: evaluation and reflection

Participant feedback from the workshop event was analysed, with participants reporting it as a positive experience, one describing it as “brilliant” (P7). When surveyed, all members of the study team reported that the event achieved its intended aims and was a positive experience. No negative impacts were reported from any participants or study team members at any stage of the process.

One study team member described that through involving people, their perceptions “significantly changed” as they learned how participants could have “valid, interesting, and often novel ideas that researchers may not have considered”. The lead investigator (PL) noted:

“What I learnt is that sometimes the researchers’ assumptions about certain things can be off or even incorrect, and that researchers can miss critical points that are important to participants and the study”.

During the process, both participants and study team members reported changed views about the value of involvement in research, demonstrating ‘transformative learning’ and co-construction of knowledge [38, 50, 51]. One member of the study team (MS) stated in the survey at the end of the process:

“I came into this project wondering how participants could be involved … I was unsure how people without a science or health background could contribute. I have learned that participants have valid, interesting, and often novel ideas that researchers may not have considered. My perceptions of involving participants in planning medical research has significantly changed”

A number of significant learning points were identified by the study team when responding to the question “do you have any advice to other researchers planning involvement for their research”. Significant learning points are summarised in Table 4.

**Table 4:** Summary of learning points from study team data

Summary of learning points from study team data	
1. Fund and prioritise involvement, make it a requirement	
2. Ethics processes take time, but can improve plans	
3. Know your audience – don’t make assumptions	
4. Value diversity in experience and knowledge	
5. A supportive team improves the experience for all	

##### Cost and value

The entire process of involving people was estimated to cost $10,000 AUD, including staff time, catering and event venue hire (see Additional File 1 for more detailed information). The value of the process was summarised by the lead investigator who stated “I learnt a lot from the process and am very glad we made the effort”.

### Section 2: perspectives of ASPREE participants and other stakeholders

Recurring themes were identified by the study team from the data sources analysed, and seven specific impacts were reported as a result of involving participants (summarised below). The analysis is divided into two sections: (1) participant views about the proposed study, (2) stakeholder views about involvement in the proposed study.

#### Participant views about the proposed study

Participants were very positive about the proposed MGRS, with one stating ‘I think it sounds very good’ [P20]. Altruism was a primary motivator for participation, with participants suggesting that outcomes of a MGRS could include benefits to themselves (personally and for their families); improving healthcare more generally; and the potential for saving lives, preventing diseases, improving quality of life, and improving future research.

Participant views on types of information they would like returned from genomic research and how the study should be funded (commercial funding versus public funding) were diverse and did not always align with the study team’s prior expectations. For example, two thirds of event participants wanted access to their own genomic data, which was described by the lead investigator as ‘very at odds with the current system’ (PL).

#### Stakeholder views about participant involvement in the proposed study

Participants were supportive about being involved, with all participants supportive of being involved by providing feedback throughout the research process (100%, 32/32), with a typical participant response being ‘I’d be happy to be involved’ [P3]. Views about enablers were shared in three of the 20 interviews coded, by all 18 of the event participants and all study team members surveyed. Views about barriers were shared in eight of the interviews with participants that were coded, and by half of the study team surveys. Further data is categorised in Table 5. Additional mapping of preferences for involvement using the STARDIT-PM tool can be found in Additional File 1 [24].

**Table 5:** Enablers and barriers for involving participants

Enablers	Quotations	Barriers	Quotations
Financial remuneration for people’s time; financial support for travel and accommodation	Running a business ‘limits me and my time’ [P15]	Living in rural areas and travel logistics a barrier to participation in face-to-face events	‘I’d like to be involved online rather than face to face because of travel difficulties’ [P6]
Learning and development opportunities for participants and researchers	‘if you tell me what would be useful, I could do it’ [P13]	Poor explanation of abstract concepts such as ‘involvement’, which can be jargonistic	‘there seemed to be confusion, or a lack of understanding of what this ‘involvement’ would actually look like’ (MS)
Small groups at events gave more people a chance to share perspectives	‘Small group’ discussions at the event ‘worked well’ (Study team member)	Self-doubt about their skills or knowledge mean they don’t think they could be involved	‘I’d probably ask a stupid question’ [P2]
Early notice of events	‘give me enough notice’ [P10]	Some people not comfortable being part of a face-to-face group	‘I get very uncomfortable in a group of people’ [P12]
Clear information about timings and time commitments, frequency of involvement and available support	Ensure ‘people are advised what’s going to happen at the workshop’ [P8]	Lack of clarity about expected time commitments	Being involved ‘depends on what’s involved and time’ [P1]
Clear information about purpose and expectations of involvement, feeling their involvement has consequences	‘What’s the endpoint – what’s the goal?’ [P11]	Unclear about what tasks they could be involved in	‘I don’t know how I could but willing to help’ [P13]
Independent facilitator when working in groups (either face-to-face or online)	I’d feel more comfortable if I had someone who was facilitating [P14]	Face to face discussions ‘dominated’ by more confident or knowledgeable people (Study team member)	‘Participants from professional backgrounds to some extent dominated some of the discussions’ (Study team member)
Short events ensure people do not get fatigued	‘any longer and I think fatigue would have dampened the enthusiasm’ (Study team member)	People feel they have limited time, are busy with work or social commitments	‘I don’t have a lot of time left in life’ [P1]
Having access and literacy in using computers and online tools	‘if I could negotiate [online discussions] I’d be happy to do that’ [P15]	Lacking access, literacy or trust in using computers, smartphones or online tools (including social media)	‘I’m hopeless with computers’ [P16], ‘I don’t have internet’ [P12]
A selection of flexible communication modes (such as face to face and facilitated online discussion forums)	‘I’d be happy to be involved – more online but if there was an occasional need to come into the city I’d be happy to do that’ [P3]	Only one mode of communication, such as expecting people to travel to events	‘online is often easier’, face to face only ‘as long as it’s not too far’ [P22], ‘travel distance is an issue’ [P20]
Involving people in research ethics and governance	‘ethics is the difference between right and wrong – you know what’s right and you don’t do what’s wrong’ [P18]	A ‘researchers know best’ attitude that doesn’t value the process of involving people (Study team member)	Researchers ‘don’t see the forest for the trees’ [P19]

The data from Table 5 is organised into themes identified during analysis, with the quotations provided as illustrative of those themes.

The lead investigator stated “the feedback has been so valuable” and that it will be “built into the design” of future research. However, the study team also identified barriers to involving people which exist for researchers. One study team member reported that at the start of the project they were “unsure how people without a science or health background” could be involved (MS). They reported a personal shift during the involvement process from not understanding how participants could be involved and being concerned about “asking too much” to believing that, “with adequate resources (financial, training, time...), people can be involved in all aspects of genomics research”.

Other barriers identified by the study team included delays in obtaining ethical approvals for involving people and the cost of involvement in both time and money. One study team member reflected in the follow-up survey that they had “worried too much” about the time-burden of involving participants (MS). The concern of not putting further pressure on participants was a theme in email communication between the study team when making decisions to limit contact with participants throughout the process of involving them.

Survey data from the study team also suggests that adequate funding, a supportive team, involving participants in the very earliest stage of research planning and co-designing inclusive methods of involvement all contributed to the impacts reported.

### Section 3: impacts on study design from stakeholder involvement

Involving stakeholders in the co-design process impacted the study in seven specific ways. By asking for participants’ views on aspects of the proposed study design, the study team gained insight into participant preferences and opinions. While there was diversity in views, the process allowed the study team to improve aspects of the study design. As a result of the process described, the ASPREE-XT investigator agreed a number of changes to the proposed study design. These proposed changes as summarised as impacts in Table 6 ‘Summary of impacts on study design’.

**Table 6:** ‘Summary of impacts on study design’

Impact on planned research	Summary of impact
**1: Recruitment and sample collection**	Recruitment and consent for the MGRS will occur online wherever possible, and saliva samples (rather than blood) will be sent by post to be used as biospecimens for DNA analysis.
**2: Participant communication**	A short video and ‘information pack’, which will explain the MGRS study, will be created to assist with recruiting family members.
**3: Participant involvement in governance**	Participants will be invited to be involved in overseeing governance, including funding decisions.
**4: Data access**	Study participants should be involved in controlling data access decisions and policies.
**5: Communication and ways of involving participants**	Participants will be included on study advisory groups, including for study recruitment and communication, data access and ethical oversight using multiple communication modes.
**6: Provide feedback to participants about the research**	Participants will be informed about the impact of the research, and how their involvement has affected the design and management of the study.
**7: Create learning and development opportunities**	Learning and development opportunities will be created for potential participants (developing knowledge of research), researchers (developing skills in involving people) and other stakeholders.

#### Impact 1: recruitment and sample collection approach

A discussion about participants’ adult children being ‘time-poor’ highlighted the importance of a study design which minimised the time burden on younger generations. Only a third of participants thought their children would be willing to do a blood test. This allowed the research team to make a more informed decision about ‘trade-offs’ between the data that can be collected via blood or saliva, versus the potential effect on recruitment.

#### Impact 2: participant communication

Most participants reported willingness to be involved in recruiting family members to a new study, if given appropriate information and supporting documents. Relatively inexpensive information resources, such explanatory videos, could have a significant impact on the success of the recruitment and the study as a whole. One participant also advised that information produced for participants by researchers can be confusing, and that laypeople can help simplifying it.

#### Impact 3: participant involvement in governance

Event participants unanimously agreed that they should be involved in all aspects of the research, whereas 11 of participants from the 20 analysed interviews thought that they should be involved in study design. One interview participant felt only researchers should be involved as they are “the qualified people” (P8). Five of the interview participants expressed the view that non-researchers are required in research as they provide an important alternative perspective.

Participants had mixed views about commercial organisations funding research. Four event participants were against it, some were ambivalent, and the majority indicated no objections. One participant was concerned about the risks posed by involvement of commercial organisations with opaque “vested interests”, asking “what is in it for them?” (P2), referring to individuals and organisations with real or perceived conflicting, competing or commercial interests. Participants suggested that involving people in governance (including funding and ethical oversight) was a way of mitigating this risk.

Study team members reflected that public funding would be preferable to commercial funding, as the responses at the event suggested that a commercially-funded study might negatively impact recruitment. As a result, the study team altered the proposed design to involve participants in governance, oversight and funding decisions.

#### Impact 4: data access

Participants shared the view that they would like different kinds of genetic information returned from the research (see Table 3), including personal medical, ancestry and pharmacogenomic results. Two-thirds of event participants wanted access to their own genomic data, and had mixed views about who else should have access. All event participants stated they were comfortable with their data being held by academics, and none were comfortable with data being held by a commercial company. However, one participant suggested not “ruling private companies out completely” from research (P9).

General practitioners (GPs) were generally trusted to access and interpret genomic data, but participants felt GPs should not have access to data that they did not. All but two event participants agreed they should exclusively control access to their own data, with those disagreeing mentioning cognitive decline as a reason for a co-managed access model.

Some participants had concerns about themselves or their biological relatives (especially offspring) finding out information they “might not want to know” (P6), or having it imposed on them.

#### Impact 5: communication and ways of involving participants

Preferences for communication mode differed between interview participants and event participants. Interview participants preferred questionnaires via post (30%, 18/59), and face-to-face events (25%, 15/59), over online questionnaires (17%, 10/59) and online discussions (8%, 5/59). Event participants suggested face-to-face meetings were helpful but only when there was an “occasional need” (P3). Participant responses also suggested that limiting face-to-face events where possible (in favour of telecommunication) may mean involvement is more inclusive.

Event participants felt certain tasks (such as reviewing information) could be done “more online” (P3). Participants spontaneously suggested using online, moderated forums and suggested that these should be hosted by trusted institutions (such as universities) rather than commercial organisations, as some ‘don’t trust’ social media companies [P17].

#### Impact 6: provide feedback to participants about the research

Event participants stated that keeping people informed about what has been learned from the study is a good way of keeping people engaged in the study and improving retention. For example, ASPREE-XT sends a regular newsletter to participants. Participants also stated that they would like to be informed about when their involvement has made a difference.

#### Impact 7: create learning and development opportunities

Participants often stated their willingness but also their uncertainty about how they could be involved. Learning and development opportunities were identified as an area of involvement by both a participant advisor and participants. Learning and development opportunities will be need to be created for different stakeholder groups’ learning needs, including developing a knowledge of the research process for research participants and developing skills in involving people and community engagement for researchers.

## Discussion

The participatory action research method gave insights into participants’ preferences that measurably impacted on the proposed study design. The improvement of the interview design using the co-design process illustrates the value of a flexible and iterative approach to involvement in a study. It should be noted that the effective involvement of ‘stakeholders’ also extends to involving all relevant staff and health professionals at all levels of an initiative, who may have important knowledge or perspectives that senior research staff do not.

Participants’ preference for being informed about both the study outcomes and the outcomes of their involvement is supported by other studies which suggest that communicating about the research regularly and sharing results may improve retention [52–54]. This is particularly relevant for those planning involvement in genomics research, which may span decades [16].

Such regular communication should also be combined with learning and development opportunities, which could also help facilitate participant involvement by ensuring that people understand the values which motivate the tasks they are being asked to be involved in [16, 55].

Participant views about data storage and access aligns with a 2015 survey by Genetic Alliance Australia, which indicated people mostly trusted universities and research institutes to use personal genetic information for research, with commercial companies least trusted [56]. Event participants’ unanimous preference for having access to their own data and a general trust for GPs to access and interpret genomic data also aligns with findings from other studies [57–59]. Despite this preference, this option is often still not offered to research participants [16].

Participants’ concerns about unintentional disclosure of data to biological relatives who ‘might not want to know’ certain information highlights the challenges of asking participants about information preferences, and recognises that this ethical decision extends beyond individuals [P6]. This issue is common to almost all ongoing and proposed genomic research studies and should be urgently addressed by all ethical frameworks which favour an individualist perspective over a collective or community perspective [58, 60].

### Study strengths

Feedback from everyone who participated in this process was positive. By asking participants their preferences, the study team gained useful insights to inform the design of the proposed study. Participants' preference for being involved in decision making about funding sources, data management and ownership, and what information to share with participants will help ensure any future study design aligns with participants’ values, ensuring the design is culturally safe and culturally competent [61]. Similarly, the participants' preference for being involved in reviewing participant information aligns with other research which suggests that involving potential participants in reviewing information can help improve recruitment [62].

The ‘transformative learning’ during the process reported from both study participants and the study team was an important impact captured by the participatory action research (PAR) method [38, 50]. The process showed that it was valuable to create regular involvement opportunities for each stakeholder. Reporting this process in a standardised way using ‘Standardised Data on Initiatives’ (STARDIT) meant that impacts such as transformative learning could be reported and that this case study can be compared to similar studies in the future [48].

### Study limitations

Because the 76 participants who responded to the original advertisement were self-selected volunteers, our findings may not necessarily be accurate for the whole ASPREE-XT cohort or statistically representative. Our sample may reflect a sub-set of individuals who feel more strongly supportive of a proposed MGRS or other issues such as data access, compared to the cohort average. However, the data from this process is still useful and valid [57].

While the response rate was small compared to the number contacted, the invitation to be part of this process was unique for ASPREE-XT participants, and therefore cannot be compared to other similar response rates to provide a normal or average expected response. While data was not gathered on why people did not respond, the response rate may have been affected by the advertisement being small and not in a prominent position in the newsletter; participants may have felt unfamiliarity with genetics and reluctant to contribute; many participants may have not read the newsletter; some participants may have become ill or may be affected by cognitive decline.

The number of interviews which were transcribed (20) and analysed was high for a case study [63]. As there is no agreement in case study literature on whether to code all or some of the data, our methodology balanced exhaustive examination of the data with the capacity bias imposed by time constraints [45, 64]. Therefore, data transcribed and coded from the interviews does not include all the data collected in the interviews.

While the mixture of written and verbal feedback at the event ensured a range of ways to give feedback, the voting process (which involved people raising their hands in front of everyone) may have given different results if it was an anonymous ballot [59]. Future research should explore and compare preferences for different methods of voting.

While an ASPREE-XT participant advisor is an author of this paper (BH), there were both ethical and practical barriers to involving other participants in this way. It is important to note that the participant advisor involved also has a professional background in genomics, so has a different background and understanding of the study, genomics and research in general, compared with others from the ASPREE-XT population.

While the process of involving people described here did not exclude people based on language [65], the original ASPREE study required a certain level of English language skills in order for people to participate in some of the cognitive assessments and public events. The process described here may therefore have excluded people who cannot read English or do not feel confident speaking in English, such as people who speak English as a second language. Participation in this process may also have been affected by cognitive issues experienced by some participants.

## Conclusions

This case study provides evidence that including participants in the design of a research study positively impacted the study design. As many research studies are negatively impacted owing to poor recruitment and retention, such evidence is increasingly important for informing involvement in future studies. The process of involving ASPREE-XT participants in the design of a new MGRS was highly valued by stakeholders, and was positively impactful for both participants and the study team. The lead investigator stated “the feedback has been so valuable” and that it will be “built into the design” of future research. Learning from the case study suggests that adequate funding, a supportive team, involving participants in the very earliest stage of research planning and co-designing inclusive methods of involvement all contributed to the impacts reported.

The process of involving people can be viewed as a learning experience for both the participants involved and study team members. The process changed participant and study team members’ views about the value of involvement. For example, one a study team member stated they ‘learned that participants have valid, interesting, and often novel ideas that researchers may not have considered’ and their ‘perceptions of involving participants in planning medical research have significantly changed’, which can be viewed as an impact of ‘transformative learning’ [38, 50]. Using a standardised reporting tool to describe the methods and impacts provides a way for learning from this case study to inform future research studies planning to involve people, including studies beyond the discipline of public health genomics.

## Supplementary Information

**Additional file 1.** Data and Analysis. Detailed description of the data sources in this case study, how they were analysed, including STARDIT reports.

**Additional file 2.** Telephone Questionnaire. Version Comparison - A comparison of the versions of the telephone questionnaire used.

**Additional file 3.** Quantitative results - A summary of the quantitative results.

**Additional file 4.** GRIPP2 report.

**Additional file 5.** STARDIT report.

## Data Availability

All relevant data has been anonymised and shared in the additional files. Monash University is storing all raw data according to the relevant ethics policies, and invites requests for more detailed data. STARDIT reports will be added to databases in the future. Additional videos of the events will be shared in the public domain.

## Abbreviations

ASPREE: ASPirin in Reducing Events in the Elderly
ASPREE-XT: ASPirin in educing Events in the Elderly Extension
MGRS: Multigenerational research study
PAR: Participatory action research
STARDIT: Standardised Data on Initiatives
