# Supplementary material for: Involving elderly research participants in the co-design of a future multi-generational cohort study
Source: Res Involv Engagem. 2021 May 3;7:23. doi: 10.1186/s40900-021-00271-4 (PMC8094476; doi:10.1186/s40900-021-00271-4)
Supplement: Supplementary file 1 — Additional file 1. Data and Analysis. Detailed description of the data sources in this case study, how they were analysed, including STARDIT reports. [file 40900_2021_271_MOESM1_ESM.pdf]

Data and Analysis: 'Involving elderly research participants in the co-design of a future multi-generational cohort study'

Jack S Nunn[1*], Merrin Sulovski[2], Jane Tiller[2], Bruce Holloway[2], Darshini Ayton[2], Paul Lacaze[2]

[1]School of Psychology and Public Health, La Trobe University, Victoria, Australia

[2]Department of Epidemiology and Preventive Medicine, School of Public Health and Preventive Medicine, Monash University, Melbourne, Australia

Corresponding author: Jack.Nunn@latrobe.edu.au

About this document

This document contains additional data relevant to the case study 'Involving elderly research participants in the co-design of a future multi-generational cohort study'. In addition it contains a more detailed description of the data sources in this case study. This document includes the preferences mapping data (STARDIT-PM), and other data about this initiative[1]. The corresponding Standardised Data on Initiatives Alpha Version (STARDIT) of the report can be found in Additional File 5.

Contents

About this document 1
Contents 1
Data mapping 2
Budget 3
Study team survey 3
STARDIT Preference Mapping (STARDIT-PM) – Alpha Version 5
References 12

Data mapping

This table summarises all the data sources used for the case study.

Data Category	Data point description
Diary	ASPREE research diary – including reflections
ASPREE Newsletter	Advert in the newsletter for all participants asking for those ‘interested in providing input on the design of possible future genetic, family and multi-generational studies’ to get in touch
Meeting records	Meetings with the study team, including notes, audio recordings and relevant emails
Email discussions	Emails about updated versions of the questionnaire May 2018 and June 2018<br>Email discussion with study team members
Reports on progress	Interview reports from MS – June 2018
Interview participant initial feedback	Interviews with participants asking for feedback on questionnaire design changes
Meeting about interviews	Discussion with MS and JN based on interviews conducted by mid-June
Interview data	Interview recordings (audio and PDF notes)<br>Interview response highlights identified by MS
Interview summary	Interview contents are summarised in a spreadsheet by MS
Meeting about event	Study team meeting about event, informed by interviews – August 2018
Interview	Email interview with MS about interviews, including early identification of themes and learning points
Event planning feedback	Feedback from participant advisor on event facilitation plan – August 2018
Pre and post event information and questions	Information and questions sent to participants before and after the event
Facilitation plan and relevant reflections	The final facilitation plan and relevant reflections in JN’s research diary
Event recording	Audio<br>Video of event<br>Short video interviews with 4 event participants
Participant feedback about event	Participant feedback about the event, including feedback forms
Email summaries of event	Email summaries about event
Notes from event	Notes from the event by MS, PL, BH and JN
Meeting notes	Meeting notes from discussion with PL and BH

Data Category	Data point description
Email discussions	Emails to study team after event about survey – mid September
Discussion about newsletter	Feedback on newsletter by study team members – late September
Final newsletter	Final newsletter sent out to event participants and other people interviewed
Budget of involvement	Budget documents
Study team interviews	Interviews carried out by email with study team members 6 months after the event - March 2019

Budget

The budget of the process is itemised below.

Item	Cost ($AUD)
Room hire and food for event	1000
Staff time (estimated)	9000
Total	10000

Study team survey

The following questions were emailed to the study team members six months after the event:

1. Please describe your tasks in the process of involving people in planning of the new ASPREE multi-generational study

2. What did you learn from the process of involving ASPREE participants in the research planning phase?

3. Please describe specifically what worked well or was useful about the way people were involved

4. Please describe specifically what did not work well or was not useful about the way people were involved

5. Were there any barriers or facilitators to conducting the involvement activities? (institutional or otherwise)

6. Do you think the involvement activity achieved its intended aim(s)?

7. Do you have any advice to other researchers planning participant involvement for their research?

8. Describe the impact you think involving people had (positive/negative - on the research, staff or participants)

9. Who do you think should influence the kind of human genomic research done in the future, and why? (e.g. the public, participants of research studies, doctors, school children, politicians etc)

10. Which stages of future genomic research should be influenced by people other than researchers (if any)? (e.g. concept planning of new studies, study design, conducting the research, presenting the results etc)

11. Other comments

STARDIT Preference Mapping (STARDIT-PM) – Alpha Version

This table uses the Alpha version of the Standardised Data on Initiatives Preference Mapping (STARDIT-PM) to categorise the data into certain areas[1].

Area (quantitative data about responses in this area)	Qualitative summary of participants' views and perspectives	Qualitative summary of study team's views and perspectives
**Views on who should be involved:**<br><br>50% (10/20) of interview participants, 100% (18/18) of event participants and three (75%, 3/4) study team members shared a view or perspective about this area.	Two participants noted that the purpose of involving people needed to be clear in order to avoid 'wasting time'.<br><br>One participant felt only researchers should be involved as they are 'the qualified people', two others stated participants should be involved as researchers only 'see it from their point of view and nobody else's', and participants bring 'new perspectives'.<br><br>One participant mentioned 'vested interests' and suggested involving participants was a way of overcoming this.<br><br>Transparency from the project about who is involved (specific professions) might help participants identify 'different directions'.	One study team member (an ASPREE participant assessor) reflected that the 'increased autonomy' of involving other staff equally (rather than just senior research staff) made them feel valued and gave the opportunity to 'think creatively' and 'engage in controversial or difficult discussions'.

**Area (quantitative data about responses in this area)**	**Qualitative summary of participants' views and perspectives**	**Qualitative summary of study team's views and perspectives**
	Event participants were unanimous that there was no aspect of the research that they should not be involved in. One participant stated that 'funding' decisions may be better being made by experts, although participants agreed they should be involved in the oversight of research funding.	
**Views on who should do which tasks:**<br><br>25% (5/20) of interview participants, 100% (18/18) of event participants and two (50%, 2/4) study team members shared a view or perspective about this area.	Participants stated they should be involved in research design. A participant commented that feedback is needed from participants. Another participant suggested that participant information can be confusing and that a layperson can have the task of simplifying it. A third participant expressed a willingness to be involved as long as the task had purpose and was not 'just for the sake of chatting'.<br><br>10 event participants said they would be interested in being involved in recruitment and communication, 7 were willing to be involved in data access decisions and 2 in ethical decisions.	The lead investigator stated that participant involvement "significantly improves the researchers' ability to make sound decisions regarding the fundamental research questions, study design, ethics and funding applications".<br><br>The participant advisor stated ,'I believe that researchers should have the dominant responsibility to plan and complete genomic research'.
**Views on modes of communication:**<br><br>65% (13/20) of interview participants, 100% (18/18) of event participants and 25% (1/4) of study	There was much variation in views and perspectives about communication mode. For example, some participants stated a preference for face-to-face discussion, while others preferred online questionnaires, commenting on documents online or joining online text-based discussion groups.	Referring to the face-to-face event, one study team member stated, 'participants really enjoyed the opportunity to be heard and put their views forward'. After the event, the lead investigator noted that the planned research 'must use mobile/internet technology'.

Area (quantitative data about responses in this area)	Qualitative summary of participants' views and perspectives	Qualitative summary of study team's views and perspectives
team members shared a view or perspective about this area.	Participants reported perceived advantages and disadvantages for each communication mode. Two thirds of event participants said that they would be happy to be involved both face-to-face and online (using computers and smartphones).<br><br>Event participants felt certain tasks (such as reviewing information) could be done 'more online', and that face-to-face meetings were helpful when there was an 'occasional need'. Online text-based discussions were stated to have advantages by 'opening up more discussion' as it gave a chance for people to reflect on other participants' views and perspectives, meaning discussion could be more in-depth.	
**Views on what methods should be used:**<br><br>15% (3/20) of interview participants, 100% (18/18) of event participants and all (100%, 4/4) study team members shared a view or perspective about this area.	One participant stated that they did not feel comfortable being part of a face-to-face group, while another described a method of being involved which was a small group conversation with a researcher leading a discussion to gather views and ideas.<br><br>Another participant suggested having information sent out which could be read, with participants providing feedback.	After the face-to-face event, most of the study team members felt that dividing the event discussion into small groups facilitated discussion and gave more people a chance to share views and perspectives. One study team member felt that asking focussed questions and requesting a show of hands was a time-efficient way to gauge perspectives.

Area (quantitative data about responses in this area)	Qualitative summary of participants' views and perspectives	Qualitative summary of study team's views and perspectives
	Event participants spontaneously suggested using an online discussion platform and shared views on what is good moderation and the advantages of online discussion, although some shared concerns about for-profit social media platforms being used.	
**Views on facilitators of involvement:**<br><br>15% (3/20) of interview participants, 100% (18/18) of event participants and 100% (4/4) of study team members shared a view or perspective about this area.	Giving people early notice of events and clear advice about the purpose and expectations were identified as important by two participants.<br><br>One participant identified 'personality' as distinct from skills and knowledge – which could be considered a facilitator if managed appropriately.<br><br>100% of event participants felt that if they were involved in recruiting participants from their immediate family, a short explanatory video would be helpful and improve their confidence in explaining the study.<br><br>When asked about support, two event participants identified it as helpful having a person act as an independent facilitator when involved in working in groups on tasks such as ethical oversight (either face-to-face or online).	One study team member felt education was essential, and that assuming a limited knowledge of a subject and explaining the basic concepts at the start of the event was important, as this appeared to support people to make informed decisions when contributing to group discussions.<br><br>Another study team member stated that adequate funding for involvement was required so that it can become 'a requirement, rather than a luxury'.<br><br>Having a lead investigator 'who valued the unique experiences of each team member and participants' was identified as a facilitator by one study team member.

Area (quantitative data about responses in this area)	Qualitative summary of participants' views and perspectives	Qualitative summary of study team's views and perspectives
**Views on barriers of involvement:**<br><br>40% (8/20) of interview participants and 50% (2/4) of the study team shared a view or perspective about this area.	Living in rural areas and other travel logistics were considered a barrier to participation in face-to-face events by a number of participants.<br><br>A lack of clarity about expected time-commitments or timing of events was identified by four participants.<br><br>Not having the skills or knowledge was identified as a barrier. One participant felt they lacked literacy in using computers and online tools.	One study team member observed after the event that they perceived participants from 'professional backgrounds' dominating group discussions, which may have inhibited others.<br><br>Another study team member stated the 'cost' of involvement in terms of time and financial commitment might be a barrier for some research projects.
**Views on what the outcome or output of the research or involvement in research could be:**<br><br>15% (3/20) of interview participants, 22% (4/18) event participants and 100% of study team members (4/4) shared a view or perspective about this area.	Participants raised the issue of wanting to know outcomes and outputs of involvement, with one seeking clarity on what the purpose of involvement was.<br><br>Participants shared many views about the outcomes of research and felt being involved in clarifying the aims of the future study was important. Involving participants in helping answer what the research 'hoped to achieve' was an outcome identified by one participant. It was stated that 'responses from participants could cause the experts to ask new questions' or lead the research in 'different directions'.	All members of the study team thought the involvement process achieved the intended aims, and that process had a positive impact. None reported negative impacts.<br><br>One study team member stated that involving participants could help researchers make decisions about 'fundamental research questions, study design, ethics and funding applications'.

Area (quantitative data about responses in this area)	Qualitative summary of participants' views and perspectives	Qualitative summary of study team's views and perspectives
	One event participant said that her preference was not to participate in research which was 'about the aggrandisement of the professor', while another stated that the most important thing to 'get right' was having clear study aims with regard to what it is trying to achieve. Interview participants suggested that their motivation for participating was altruistic, in the hope that the research would contribute to positive outcomes for future generations.	
**Views on which stage of the research people should be involved:**<br><br>10% (2/20) of interview participants, 100% (18/18) of event participants and 100% (4/4) of study team members shared a view or perspective about this area.	Most participants agreed that people other than researchers should be involved in research design, including designing the research question. A participant commented that feedback is needed from participants.	One study team member stated that 'participant involvement is vital, especially in the early stages' of research, with 'less involvement' needed in executing the study, collecting data and analysing results. Participants could then be more involved in the 'publishing and communication' of results.
**Research data:**<br><br>0% (0/20) of interview participants, 100% of event participants (18/18) and 25% of study team members (1/4) shared a view or perspective about this area.	Seven event participants said they would be interested in being involved in decisions about data access. 100% of event participants were comfortable with their data being held by academics. 100% were not comfortable with it being held by a for-profit company, although one participant said not to 'rule private companies out completely'. All event participants were interested in having pharmacogenomic results returned. Two thirds	During the event, the lead investigator noted that while participants overwhelmingly wanted 'a self-managed future of health information', this was 'at odds' with the current healthcare professional managed information paradigm.

Area (quantitative data about responses in this area)	Qualitative summary of participants' views and perspectives	Qualitative summary of study team's views and perspectives
	of event participants wanted access to their own genomic data, and had mixed views about who else should have access. GPs were generally trusted to access and interpret genomic data, but participants felt GPs shouldn't have access to data that they did not. All but 2 participants agreed they should have access to their own data, with those disagreeing mentioning cognitive decline as a reason for a co-managed model. Some participants had concerns about them or their relatives (especially offspring) finding out information they 'might not want to know'. Questions about duty of disclosure and how this might affect 'employability' were also asked, with participants seeking clarity about how these issues would affect the research design regarding return of data.	

References

1. Nunn JS, Shafee T, Chang S, et al. Standardised Data on Initiatives - STARDIT: Alpha Version. 2019. doi:10.31219/OSF.IO/5Q47H
