# Supplementary material for: Involving elderly research participants in the co-design of a future multi-generational cohort study
Source: Res Involv Engagem. 2021 May 3;7:23. doi: 10.1186/s40900-021-00271-4 (PMC8094476; doi:10.1186/s40900-021-00271-4)
Supplement: Supplementary file 4 — Additional file 4. GRIPP2 report. [file 40900_2021_271_MOESM4_ESM.pdf]

GRIPP2 report for ‘Involving elderly research participants in the co-design of a future multi-generational cohort study’

This report has been completed using the ‘GRIPP2 reporting checklists: tools to improve reporting of patient and public involvement in research’ available at https://doi.org/10.1136/bmj.j3453.

**Category**	**Category description**	**Data**
1a: Aim	Report the aim of the study	Participatory action research to involve elderly research participants in the co-design of a proposed multi-generational cohort study, in order to improve research design, relevance, acceptability and recruitment.
1b: Methods	Describe the methods used by which patients and the public were involved	Participatory action research to involve elderly research participants in the co-design of a proposed multi-generational cohort study.
1c: Results	Report the impacts and outcomes of PPI in the study	Improved participant information resources, improved wording that is culturally appropriate, improved question design for interviews, improved learning resources for participants, improved co-design process.
1d:Conclusions	Summarise the main conclusions of the study	Involving participants in co-designing a proposed study resulted in changes to the design of the proposed study<br>The process of involving people can be viewed as a learning experience for both the participants involved and study team members. The process changed participant and study team members’ views about the value of involvement, which can be viewed as an impact of ‘transformative learning’.
1e: Keywords	Include PPI, “patient and public involvement,” or alternative terms as keywords	Public Health; Epidemiology; Preventive Medicine; Medical Ethics; Medical Education & Training; public involvement; participatory research; genomics; patient involvement;

Category	Category description	Data
2a: Definition	Report the definition of PPI used in the study and how it links to comparable studies	The words ‘involvement’ or ‘being involved’ describe the concept of people being ‘involved’ in research. This is when research is carried out ‘with’ people rather than ‘on’ them. ‘Involvement’ can also be defined as when other people aside from the research team, such as the public, patients, research participants and other stakeholders, actively contribute to the research process. It is the ‘active involvement’ in shaping and guiding research, rather than only providing data.
2b: Theoretical underpinnings	Report the theoretical rationale and any theoretical influences relating to PPI in the study	The process was guided by a number of international participatory action research methodology frameworks, including the International Collaboration for Participatory Health Research and INVOLVE guidance on co-design. An Alpha version of ‘Standardised Data on Initiatives’ (STARDIT) was used to map people’s preferences for involvement in a standardised way, including mapping views on who should be involved and how. STARDIT was then used to guide co-design of the process, and to subsequently report how people were involved, using standardised data.
2c: Concepts and theory development	Report any conceptual or theoretical models, or influences, used in the study	We used a case study research methodology to record and describe the process of involving participants in the co-design. The process was guided by a number of international participatory action research methodology frameworks, including the International Collaboration for Participatory Health Research and INVOLVE guidance on co-design. An Alpha version of ‘Standardised Data on Initiatives’ (STARDIT) was used to map people’s preferences for involvement in a standardised way, including mapping views on who should be involved and how. STARDIT was then used to guide co-design of the process, and to subsequently report how people were involved, using standardised data.
3: Aim	Report the aim of the study	Participatory action research to involve elderly research participants in the co-design of a proposed multi-generational cohort study, in order to improve research design, relevance, acceptability and recruitment.
4a: Design	Provide a clear description of methods by which patients and the public were involved	The study team held four meetings to co-design the involvement activities. One participant advisor was involved in a number of tasks including reviewing and improving the written information, telephone interview questions, and the facilitation plan for the event.<br>After the recruitment and consent process, participants were interviewed by telephone. Participants were asked about their willingness to provide feedback throughout the study, and to be involved in study design, as well as preferences for modes of communication.

Category	Category description	Data
		Eighteen participants attended a four-hour workshop event in central Melbourne. The event was co-designed by the study team, and was informed by interview data and international best-practices for involvement events.
4b: People involved	Provide a description of patients, carers, and the public involved with the PPI activity in the study	3 academic research investigators<br>An ASPREE participant assessor<br>An ASPREE-XT participant
4c: Stages of involvement	Report on how PPI is used at different stages of the study	Stage 1: Planning<br><br>The study team held four meetings to co-design the involvement activities. One participant advisor was involved in a number of tasks including reviewing and improving the written information, telephone interview questions, and the facilitation plan for the event.<br><br>Stage 2: Recruitment and telephone interviews<br><br>An advert was placed in a newsletter to 14,268 ASPREE participants. After the recruitment and consent process, participants were interviewed by telephone. Participants were asked about their willingness to provide feedback throughout the study, and to be involved in study design, as well as preferences for modes of communication. The definition of involvement below used in the script was co-designed with participants for subsequent interviewees.<br><br>Stage 3: Event<br>Eighteen participants attended a four-hour workshop event in central Melbourne. The event was co-designed by the study team, and was informed by interview data and international best-practices for involvement events.<br><br>The event included an introduction to the proposed MGRS by the lead ASPREE-XT genomics researcher (PL); a plain-English introduction to genomics by an expert in genomics who is also an ASPREE-XT participant (BH); a summary of the telephone interview results by the interviewer (MS); and an

Category	Category description	Data
		interactive session which included open questions about the types of information participants would like returned and recruitment of family members.<br>The final session included a presentation and interactive discussion about involvement in research, led by the event facilitator (Jack Nunn). This session explored preferences about how people would like to be involved, with open and closed questions. Questions included preferences about tasks and modes of communication.<br><br>Throughout the event, participants shared their views on a range of issues through interactive discussions, voting (by show of hands) and anonymous written feedback.<br>Stage 4: Evaluation and analysis<br>Members of the study team were surveyed six months after the face-to-face event in order to integrate the valuable views and perspectives of those involved in co-designing and delivering the process. Design of surveys was informed by frameworks for planning and reporting public involvement (GRIPP2 and PiiAF). The study team were asked 11 questions and the data from the four interviews was coded and categorised using Standardised Data on Initiatives (STARDIT).<br><br>The stages of qualitative data analysis included data mapping and familiarisation; transcription; coding; searching for themes; reviewing themes with study team members (including a participant representative); labelling and summarising themes; and reporting the findings. In order to enhance validity of the analysis, two authors independently analysed the data thematically, which was then ‘member checked’ by a third author. Standardised categories (STARDIT) were used during content analysis of the data in order to facilitate comparison with other research projects. More information about the data sources and a STARDIT report available.
4d: Level or nature of involvement	Report the level or nature of PPI used at various stages of the study	Participants were involved at every level of every stage, with more information in section 4C. Everyone listed in 4B was involved in co-designing every stage of the process. This included refining wording of participant information, sharing views and advice about the process, proof-reading documents, providing feedback on questionnaires, analysing data, informing planning, presenting information to participants, interpreting data, and participating in email surveys.

Category	Category description	Data
5a: Qualitative evidence of impact	If applicable, report the methods used to qualitatively explore the impact of PPI in the study	The stages of qualitative data analysis included data mapping and familiarisation; transcription; coding; searching for themes; reviewing themes with study team members; labelling and summarising themes; and reporting the findings. In order to enhance validity of the analysis, two authors independently analysed the data thematically, which was then 'member checked' by a third author. Standardised categories (STARDIT) were used during content analysis of the data in order to facilitate comparison with other research projects.<br><br>Involving stakeholders in the co-design process impacted the study in seven specific impacts ways. By asking for participants' views on aspects of the proposed study design, the study team gained insight into participant preferences and opinions. While there was diversity in views, the process allowed the study team to improve aspects of the study design.
5b: Quantitative evidence of impact	If applicable, report the methods used to quantitatively measure or assess the impact of PPI	Twenty relevant interviews were transcribed, coded and categorised, with relevant interviews identified by two investigators independently. To reduce any unconscious selection bias, a sample of over 10% of the interviews was selected at random.
5c: Robustness of measure	If applicable, report the rigour of the method used to capture or measure the impact of PPI	We used the 'Standardised Data on Initiatives (STARDIT)' Alpha Version to plan and report how participant involvement activities positively impacted the study design. STARDIT includes a tool to map people's preferences for involvement in a standardised way, including mapping views on who should be involved and how. STARDIT was then used to guide co-design of the process, and to subsequently report how people were involved, using standardised data. An Alpha version of the STARDIT framework was also used in parallel with the thematic analysis to organise data into pre-defined 'super-categories' which allow consistent comparison with other data using this reporting framework.
6: Economic assessment	If applicable, report the method used for an economic assessment of PPI	The entire process of involving people was estimated to cost $10,000 AUD, including staff time, catering and event venue hire. The value of the process was summarised by the lead investigator who stated "I learnt a lot from the process and am very glad we made the effort".
7a: Outcomes of PPI	Report the results of PPI in the study, including both	Improved participant information resources, improved wording that is culturally appropriate, improved question design for interviews, improved learning resources for participants, improved co-design process.

Category	Category description	Data
	positive and negative outcomes	
7b: Impacts of PPI	Report the positive and negative impacts that PPI has had on the research, the individuals involved (including patients and researchers), and wider impacts	Involving stakeholders in the co-design process impacted the study in seven specific impacts ways. By asking for participants' views on aspects of the proposed study design, the study team gained insight into participant preferences and opinions. While there was diversity in views, the process allowed the study team to improve aspects of the study design.<br><br>1: Recruitment and sample collection<br>Recruitment and consent for the MGRS will occur online wherever possible, and salvia samples (rather than blood) will sent by post to be used as biospecimens for DNA analysis.<br><br>2: Participant communication<br>A short video and 'information pack', which will explain the MGRS study, will be created to assist with recruiting family members.<br><br>3: Participant involvement in governance<br>Participants will be invited to be involved in overseeing governance, including funding decisions.<br><br>4: Data access<br>Study participants should be involved in controlling data access decisions and policies.<br><br>5: Communication and ways of involving participants<br>Participants will be included on study advisory groups, including for study recruitment and communication, data access and ethical oversight using multiple communication modes.<br><br>6: Provide feedback to participants about the research<br>Participants will be informed about the impact of the research, and how their involvement has affected the design and management of the study.<br><br>7: Create learning and development opportunities

Category	Category description	Data
		Learning and development opportunities will be created for potential participants, researchers and other stakeholders.
7c: Context of PPI	Report the influence of any contextual factors that enabled or hindered the process or impact of PPI	The process took longer than expected. There is confusion over what ethics approval is required in order to involve people, especially people who are participants in an ongoing study.<br><br>Involving field staff (as well as senior researchers and academics) provided a valuable perspective, as some staff knew some participants personally and had knowledge that senior research staff did not.<br><br>Some study team members worried about over-burdening participants by asking them to do too much, however this concern did not seem to be backed up by the data collected, and may be considered a barrier to involvement.<br><br>Enablers of involvement - Giving people time to read resources. Clear communication about the intention of involving people.<br><br>Barriers of involvement - Face-to-face meetings were difficult to organise. Some participants were elderly or lived in remote areas, so face-to-face meetings needed to be minimised where possible.
7d: Process of PPI	Report the influence of any process factors, that enabled or hindered the impact of PPI	The process took longer than expected. There is confusion over what ethics approval is required in order to involve people, especially people who are participants in an ongoing study.<br><br>Involving field staff (as well as senior researchers and academics) provided a valuable perspective, as some staff knew some participants personally and had knowledge that senior research staff did not.<br><br>Some study team members worried about over-burdening participants by asking them to do too much, however this concern did not seem to be backed up by the data collected, and may be considered a barrier to involvement.

Category	Category description	Data
7ei: Theory development	Report any conceptual or theoretical development in PPI that have emerged	The effective involvement of 'stakeholders' also includes involving all relevant staff and health professionals at all levels of an initiative, who may have important knowledge or perspectives that senior research staff do not. In other words, the 'PPI' label for this question does not incorporate all relevant stakeholders who are not described by this acronym.<br>The participatory action research method gave insights into participants' preferences that measurably impacted on the proposed study design. The improvement of the interview design using the co-design process illustrates the value of a flexible and iterative approach to involvement in a study.<br>By asking participants their preferences, the study team gained useful insights to inform the design of the proposed study. Participants preference for being involved in decision making about funding sources, data management and ownership, and what information to share with participants will help ensure any future study design aligns with participants' values, ensuring the design is culturally safe and culturally competent
7eii: Theory development	Report evaluation of theoretical models, if any	The 'transformative learning' during the process reported from both study participants and the study team was an important impact captured by the participatory action research (PAR) method. The process showed that it was valuable to create regular involvement opportunities for each stakeholder. Reporting this process in a standardised way using 'Standardised Data on Initiatives' (STARDIT) meant that impacts such as transformative learning could be reported and that this case study can be compared to similar studies in the future.
7f: Measurement	If applicable, report all aspects of instrument development and testing (eg, validity, reliability, feasibility, acceptability, responsiveness, interpretability, appropriateness, precision)	'Standardised Data on Initiatives' (STARDIT)was used to map people's preferences for involvement in a standardised way, including mapping views on who should be involved and how. STARDIT was then used to guide co-design of the process, and to subsequently report how people were involved, using standardised data. An Alpha version of the STARDIT framework was also used in parallel with the thematic analysis to organise data into pre-defined 'super-categories' which allow consistent comparison with other data using this reporting framework.
7 g: Economic assessment	Report any information on the costs or benefit of PPI	The entire process of involving people was estimated to cost $10,000 AUD, including staff time, catering and event venue hire. The value of the process was summarised by the lead investigator who stated "I learnt a lot from the process and am very glad we made the effort".

Category	Category description	Data
8a: Outcomes	Comment on how PPI influenced the study overall. Describe positive and negative effects	No negative impacts were reported from any participants or study team members at any stage of the process. Involving stakeholders in the co-design process impacted the study in seven specific impacts ways. By asking for participants' views on aspects of the proposed study design, the study team gained insight into participant preferences and opinions. While there was diversity in views, the process allowed the study team to improve aspects of the study design. Outcomes included Improved participant information resources, improved wording that is culturally appropriate, improved question design for interviews, improved learning resources for participants, improved co-design process.
8b: Impacts	Comment on the different impacts of PPI identified in this study and how they contribute to new knowledge	Involving stakeholders in the co-design process impacted the study in seven specific impacts ways. By asking for participants' views on aspects of the proposed study design, the study team gained insight into participant preferences and opinions. While there was diversity in views, the process allowed the study team to improve aspects of the study design.<br><br>1: Recruitment and sample collection<br>Recruitment and consent for the MGRS will occur online wherever possible, and salvia samples (rather than blood) will sent by post to be used as biospecimens for DNA analysis.<br><br>2: Participant communication<br>A short video and 'information pack', which will explain the MGRS study, will be created to assist with recruiting family members.<br><br>3: Participant involvement in governance<br>Participants will be invited to be involved in overseeing governance, including funding decisions.<br><br>4: Data access<br>Study participants should be involved in controlling data access decisions and policies.<br><br>5: Communication and ways of involving participants<br>Participants will be included on study advisory groups, including for study recruitment and communication, data access and ethical oversight using multiple communication modes.

Category	Category description	Data
		6: Provide feedback to participants about the research<br>Participants will be informed about the impact of the research, and how their involvement has affected the design and management of the study.<br><br>7: Create learning and development opportunities<br>Learning and development opportunities will be created for potential participants, researchers and other stakeholders.
8c: Definition	Comment on the definition of PPI used (reported in the Background section) and whether or not you would suggest any changes	The acronym ‘patient public involvement’ here is limiting, as it does not incorporate research participants and other stakeholders such as study staff, who might have unique insights into study design. The words ‘involvement’ or ‘being involved’ describe the concept of people being ‘involved’ in research. This is when research is carried out ‘with’ people rather than ‘on’ them. ‘Involvement’ can also be defined as when other people aside from the research team, such as the public, patients, research participants and other stakeholders, actively contribute to the research process. It is the ‘active involvement’ in shaping and guiding research, rather than only providing data.
8d: Theoretical underpinnings	Comment on any way your study adds to the theoretical development of PPI	An Alpha version of ‘Standardised Data on Initiatives’ (STARDIT) was used to map people’s preferences for involvement in a standardised way, including mapping views on who should be involved and how. STARDIT was then used to guide co-design of the process, and to subsequently report how people were involved, using standardised data. STARDIT includes a tool to map people’s preferences for involvement in a standardised way, including mapping views on who should be involved and how. An Alpha version of the STARDIT framework was also used in parallel with the thematic analysis to organise data into pre-defined ‘super-categories’ which allow consistent comparison with other data using this reporting framework. Creating consistency in terminology to describe the ‘who’, ‘what’ and ‘how’ of involvement allows better comparison.
8e: Context	Comment on how context factors influenced PPI in the study	The process took longer than expected. There is confusion over what ethics approval is required in order to involve people, especially people who are participants in an ongoing study.<br><br>Involving field staff (as well as senior researchers and academics) provided a valuable perspective, as some staff knew some participants personally and had knowledge that senior research staff did not.

Category	Category description	Data
		Some study team members worried about over-burdening participants by asking them to do too much, however this concern did not seem to be backed up by the data collected, and may be considered a barrier to involvement.
8f: Process	Comment on how process factors influenced PPI in the study	During the process, both participants and study team members reported changed views about the value of involvement in research, demonstrating ‘transformative learning’ and co-construction of knowledge.
8 g: Measurement and capture of PPI impact	If applicable, comment on how well PPI impact was evaluated or measured in the study	Detailed data was collected and shared. Participants were supportive about being involved, with all participants supportive of being involved by providing feedback throughout the research process (100%, 32/32), with a typical participant response being ‘I’d be happy to be involved’. Views about enablers were shared in three of the 20 interviews coded, by all 18 of the event participants and all study team members surveyed. Views about barriers were shared in eight of the interviews coded and by half of the study team surveys. Mapping of preferences for involvement was completed using the STARDIT-PM tool, with the involvement reported using STARDIT Alpha.
8 h: Economic assessment	If applicable, discuss any aspects of the economic cost or benefit of PPI, particularly any suggestions for future economic modelling.	Economic assessment needs to be widened to ‘assessing value’, with one way of this being measured being financial. The entire process of involving people was estimated to cost $10,000 AUD, including staff time, catering and event venue hire. The value of the process was summarised by the lead investigator who stated “I learnt a lot from the process and am very glad we made the effort”.
8i: Reflections/critical perspective	Comment critically on the study, reflecting on the things that went well and those that did not, so that others can learn from this study	A number of significant learning points were identified by the study team when responding to the question “do you have any advice to other researchers planning involvement for their research”. Significant learning points are were:<br>1. Fund and prioritise involvement, make it a requirement<br>2. Ethics processes take time, but can improve plans<br>3. Know your audience – don’t make assumptions<br>4. Value diversity in experience and knowledge<br>5. A supportive team improves the experience for all
