# Supplementary material for: Involving elderly research participants in the co-design of a future multi-generational cohort study
Source: Res Involv Engagem. 2021 May 3;7:23. doi: 10.1186/s40900-021-00271-4 (PMC8094476; doi:10.1186/s40900-021-00271-4)
Supplement: Supplementary file 5 — Additional file 5. STARDIT report. [file 40900_2021_271_MOESM5_ESM.pdf]

Standardised Data on Initiatives (STARDIT) report: ‘Involving elderly research participants in the co-design of a future multi-generational cohort study’

About this report

This report uses the Standardised Data on Initiatives Alpha version (STARDIT)[1].

STARDIT Report Alpha Version

**Identifying information**	
Initiative name	Involving ASPREE-XT participants in co-design of a future multi-generational cohort study
Geographic location or scope	Australia
Date range (planned start and end dates of initiative)	2017-2019
Purpose of the initiative	Participatory action research to involve elderly research participants in the co-design of a proposed multi-generational cohort study, in order to improve research design, relevance, acceptability and recruitment.
Organisations or other initiatives involved (list all if multi-centre)	1. Department of Epidemiology and Preventive Medicine, School of Public Health and Preventive Medicine, Monash University<br>2. School of Psychology and Public Health, La Trobe University
Funding sources	Department of Epidemiology and Preventive Medicine, School of Public Health and Preventive Medicine, Monash University
Clinical trial registration details (if applicable)	https://clinicaltrials.gov/show/NCT01038583
Ethics approval (if applicable)	Monash University
Other relevant information (free text)	This report describes involving potential participants in co-designing a proposed multi-generational research study. It would recruit participants from the existing ASPREE-XT study.
At which stage of the research project has this report been written? (Select from:	**After** the co-design process occurred, but before the proposed multi-generational research study has been approved or funded.

1. **Before** the intervention or initiative– this report is prospective or describes planned activity<br>2. **Ongoing** – the intervention or initiative is still taking place<br>3. **After** the research project or initiative has occurred	
Methods of the initiative (what is planned to be done, or is being reported as done)	Participatory action research to involve elderly research participants in the co-design of a proposed multi-generational cohort study.
**Report authorship**	
Name	Jack Nunn
Public domain profiles, institutional pages	https://scholars.latrobe.edu.au/display/j2nunn
Open Researcher and Contributor ID (orcid.org)	https://orcid.org/0000-0003-0316-3254
Tasks in report completion	Main author
Date of report authorship	22nd July 2020
Key contact at initiative for confirming report content	Paul Lacaze, PhD, Head, Public Health Genomics Program, Paul.Lacaze@monash.edu
**Involvement**	
Who was involved	1. 3 academic research investigators<br>2. An ASPREE participant assessor<br>3. An ASPREE-XT participant
Specific tasks of this person or group (list as many as possible) – *including any information about why certain people were included or excluded in certain tasks*	Everyone listed above was involved in co-designing every stage of the process. This included refining wording of participant information, sharing views and advice about the process, proof-reading documents, providing feedback on questionnaires, analysing data, informing planning, presenting information to participants, interpreting data, and participating in email surveys.
How were these people involved (what methods were used)	Face to face meetings, email communication, shared online documents, teleconferences.
Enablers of involvement (what do you expect will help these people get involved – or what helped them get involved)	Giving people time to read resources. Clear communication about the intention of involving people.
Barriers of involvement (what do you expect will inhibit these people from getting involved – or what inhibited them from getting involved). Are there any known equity issues which may contribute?	Face-to-face meetings were difficult to organise. Some participants and participant representatives were elderly or lived in remote areas, so face-to-face meetings needed to be minimised where possible.

What was the outcome or output of the involvement of these people? What changed as a result of involving people?	Improved participant information resources, improved wording that is culturally appropriate, improved question design for interviews, improved learning resources for participants, improved co-design process.
Which stage of the initiative were these people involved? (select from list of pre-defined stages or allow 'other')	All stages
What was the estimated financial cost for involving people. How much time did it take. Were there any costs that cannot be measured financially?	$10,000 AUD was the estimated cost for the process. The total number of hours, including staff time was estimated to be around 200, including telephone interviews and excluding data analysis.<br>Some people who attended events were unable to work or care for people on that day, and these costs were not calculated.
What worked well, what could have been improved? Was anything learned from the process of involving these people?	The process took longer than expected. There was confusion over what ethics approval was required in order to involve people, especially people who are participants in an ongoing study.<br><br>Involving field staff (as well as senior researchers and academics) provided a valuable perspective, as some staff knew some participants personally and had knowledge that senior research staff did not.<br><br>Some study team members worried about over-burdening participants by asking them to do too much, however this concern did not seem to be backed up by the data collected, and may be considered a barrier to involvement.
**Mapping financial or other 'interests'**	
Describe any financial relationship or other interest this person has to this project	Three members of the study team were employed by Monash University during this process.
Describe any conflicting or competing interests	N/A
**Data**	
Who is the data from this intervention shared with?	It will be published open access
How is it stored and hosted?	It will be shared on a public domain repository.
Who is analysing the data?	The study team described above
What methods will be used to analyse the data (including a link to any relevant code and information about validity)	We used case study methodology to describe our experience involving participants in the co-design of the proposed study. We collected and analysed both qualitative and quantitative data during the involvement activities.

	We analysed data from audio recordings of interviews and events, meeting notes, emails, reflexive diary entries and survey responses of study investigators. Coding and thematic analysis of qualitative data was carried out by two authors independently and checked by other authors.
How is information about this data disseminated?	1. It will be published in an open access journal<br>2. It will be shared as an item in a newsletter to participants of the ASPREE-XT study<br>3. Learning from this process will be presented at conferences, shared on social media and through other channels (such as podcasts).
Who 'owns' the data or claims any kind of 'intellectual property' (include relevant licensing information)	Monash University
Who controls access to the data	Monash University
How is/will the data be 'Findable, Accessible, Interoperable, Reusable' according to the FAIR criteria?	Data will be shared in the public domain and licensed under a Creative Commons license.
**Impacts and outcomes**	
What new knowledge has been generated? (if appropriate, include effect size, relevant statistics and level or evidence)	1. Involving participants in co-designing a proposed study resulted in changes to the design of the proposed study<br>2. The process of involving people can be viewed as a learning experience for both the participants involved and study team members. The process changed participant and study team members' views about the value of involvement, which can be viewed as an impact of 'transformative learning'.
Describe how the learning or knowledge generated from this initiative has or will be used	1. Knowledge from this process will inform the design of a future multi-generational study<br>2. Learning from this process can inform future involvement activities
How has or how will this be measured?	Future STARDIT reports
Who is involved in measuring this?	The study team

References

1. Nunn JS, Shafee T, Chang S, et al. Standardised Data on Initiatives - STARDIT: Alpha Version. 2019. doi:10.31219/OSF.IO/5Q47H

Corresponding author: Jack.Nunn@latrobe.edu.au

http://orcid.org/0000-0003-0316-3254
